# Does Digital Inclusive Finance Effectively Promote Agricultural Green Development?—A Case Study of China

**DOI:** 10.3390/ijerph19126982

**Published:** 2022-06-07

**Authors:** Hua Guo, Fan Gu, Yanling Peng, Xin Deng, Lili Guo

**Affiliations:** College of Economics, Sichuan Agricultural University, Chengdu 611130, China; 14102@sicau.edu.cn (H.G.); summergf0412@163.com (F.G.); jxyanling@163.com (Y.P.); dengxin@sicau.edu.cn (X.D.)

**Keywords:** digital inclusive finance, agricultural green development, dynamic spatial Durbin model, entropy-weighted TOPSIS method, sustainable development, China

## Abstract

Agricultural green development is increasingly being discussed in sustainable development. This paper constructs agricultural green development from four dimensions: resource savings, environmental protection, ecological conservation, and quality industrialization. We apply the entropy-weighted Technique for Order Preference by Similarity to an Ideal Solution (TOPSIS) method to measure agricultural green development and employ a panel dataset of provinces in China from 2011–2019. Then, the dynamic spatial Durbin model is adopted to estimate the spatial effect of digital inclusive finance on agricultural green development. The main findings are as follows: (1) digital inclusive finance effectively promotes agricultural green development, and the promotional effect shows temporary and spatial spillover; (2) regional heterogeneity exists in the spatial effect in the short and long term; and (3) education, digital infrastructure, and traditional finance are important factors influencing this spatial effect of digital inclusive finance on agricultural green development.

## 1. Instruction

Agricultural green development is an important concept within the 2030 Sustainable Development Goals proposed by the United Nations [1]. 2019 data shows that China produces 21% of global food needs and feeds 18% of the world’s population on only 8% of limited cropland; however, behind the high production of Chinese food is the consumption of chemical fertilizer and pesticides, with 35% and 42% of the world’s total, respectively (original data form: https://www.fao.org/faostat/en/?#data, accessed on 1 June 2022). With the improvement of living standards, the Chinese have paid more attention to food safety and environmental protection [2]. Agricultural green development is devoted to practicing the protection of the agricultural environment in production, sales, and consumption, alleviating the conflicts among people, land, and the environment [3]. From the perspective of economics, agricultural green development has positive externalities to social development [4], so the government needs to provide more legal and financial support for agricultural green development. Meanwhile, financial markets are reluctant to enter rural areas, leading to a lack of agricultural investment [5]. Therefore, insufficient investment has severely hindered sustainable development in agriculture.

According to the 49th Statistical Report on China’s Internet Development, 284 million rural residents have access to the Internet in 2021. Digital inclusive finance, which through digital technologies provides accessible and affordable financial services and products for Chinese farmers cannot be completely covered by traditional finance [6], but can play a significant role in alleviating the financial constraints of agricultural green development. The possible channels are shown as follows. First, digital inclusive finance, which relies on big data technology and product innovation, can not only broaden service coverage by the Internet but also reduce the transaction costs of financial products [7]. This effectively solves the long-standing problems of scattered demand subjects and insufficient effective supply in the rural financial market [8], improving the availability of financial services for farmers and increasing agricultural investment. Next, with increasing agricultural investment, more and more agricultural machinery will be put into agricultural production [9], which is conducive to solving the long-standing problem of a lack of agricultural input for most farmers. In addition, digital inclusive finance can also improve digitization in the agricultural chain [10], promoting the cross-regional flow of agricultural resources and improving their efficiency. Second, the diversification of finance services can be improved by digital inclusive finance [11]. As asserted by Yu and Wang [12], the creation of more personal kinds of agricultural insurance can reduce business risks and secure farmers’ interests. Furthermore, digital inclusive finance, when combined with information technology, can enhance farmers’ credit by innovating on the forms of collateral, such as farmland, houses, and social relations [13]. Third, digital inclusive finance can boost entrepreneurship among farmers to increase their income [14,15]. It can also advance the competitiveness of agricultural products and the export rate of agricultural products to increase farmers’ agricultural income by, for example, establishing the traceability of green production and green certificates. As financial products become more accessible, the effective utilization rate of idle funds increases, and farmers’ non-agricultural income also increases [16].

Furthermore, Li et al. [17] found that digital inclusive finance has a spatial spillover effect. We take spatial characteristics into consideration as an important research perspective.

This paper estimates the level of agricultural green development in 31 Chinese provinces by employing the entropy-weighted TOPSIS method. Base on this, the dynamic spatial Durbin model is used to explore the effect of digital inclusive finance on agricultural green development. Then, this paper chooses education, digital infrastructure, and rural traditional finance as moderating variables to discuss the effect in depth. The marginal contribution of this article includes two main aspects. First, to the best of our knowledge, no study has focused on the effect of digital inclusive finance on agricultural green development. This paper extends the existing literature by discussing the spatial impact. Second, this article explores the moderating effect of education, digital infrastructure, and rural traditional finance when the spatial effect of digital inclusive finance on agricultural green development is studied.

The remainder of the paper is organized as follows: Section 2 presents the reviews of agricultural green development and digital inclusive finance in China, Section 3 presents the materials and methods, Section 4 introduces the econometric model, Section 5 analyzes the empirical results, and Section 6 provides the conclusions and policy implications.

## 2. Review of the Literature

### 2.1. Agricultural Green Development

To empirically analyze the effect of digital inclusive finance on agricultural green development, we must first clarify what agricultural green development is and how it is measured. The present understanding of agricultural green development is divided into two stages. The first stage constitutes the greening of the agricultural production mode, which emphasizes the reduction of pesticides, fertilizers, and plastic-films [18]. The second stage focuses on the principles of sustainability and believes that agricultural pollution will affect the environment through the ecological cycle. Aceleanu [19] defined agricultural green development as the path for agriculture to realize the coordinated development of economic, social, environmental, and ecological benefits. Struik and Kuyper [20] believed that, on the premise of ensuring sufficient food and supporting the growing population, agricultural green development is a process of pursuing low pollution and high efficiency. Bhatnagar and Poonia [21] put forward the notion that agricultural green development is a compound definition with green development as the concept, sustainable agricultural development as the goal, and model of ecological agriculture as the means.

The current understanding of agricultural green development has different forms of measurement. Xu et al. [22], constructed an indicator system including labor, land, machinery, fertilizer, pesticide, plastic film, and irrigation to measure green total factor productivity, also known as the entropy weight method. Kasztelan and Nowak [23] measured agricultural green development within the framework of sustainable development, using the entropy weight method measured directly by agricultural resource indicators such as land, water, and pesticides. Wang et al. [24] followed a new perspective of agroecosystems and used the weighted average method alongside the concept of agricultural green development of green production, green innovation, green eco-protection, and green economy.

Under the premise of determining how to measure agricultural green development in China, existing studies have analyzed its level and characteristics. For example, Zhang and Chen [25] found that the overall level of agricultural green development was steadily increasing. Deng et al. [26] argued that its level shows a trend of rising first and then falling. Guo et al. [27] noted that from 2007 to 2018, the level of agricultural green development in China shows the regional heterogeneity of east > middle > west. Studies by Chen et al. [28] and Chen et al. [29] showed that agricultural green development has continuity and significant positive spatial correlation. Further, some studies have also examined the influence of factors such as traditional finance and agricultural insurance [30], internet development [31], agricultural mechanization [32], and etc. In the European Union (EU), the government interventionism has gradually brought the greening of traditional agriculture to the forefront [33]. At the same time, payment for organic agriculture is also very important for agricultural green development [34].

### 2.2. Digital Inclusive Finance in China

The World Bank and the G20 have vigorously promoted the development of inclusive finance in developing countries since 2010. With the population of the Internet and mobile devices, China seized the opportunity to promote the integrated development of inclusive finance and digital technology and pioneered the concept of digital inclusive finance. The 2016 G20 summit in Hangzhou, China took digital inclusive finance as the important topic, and put green finance into the agenda for the first time. According to the actual situation of China’s digital inclusive finance development, Peking University released the digital inclusive finance index from 2011 to 2020, providing the statistical data for scholars’ research.

China’s digital inclusive finance has achieved rapid development, establishing itself as a cutting-edge world leader in this space [35]. Meanwhile, it has also experienced regional convergence, gradually narrowing the differences between regions [36]. Furthermore, digital inclusive finance suggests strong spatial agglomeration and spatial spillover [37]. The former shows that the development level of digital inclusive finance in the eastern region is high, while that in the middle and western regions is relatively lower. The latter suggests that the development of digital inclusive finance in a province will have an impact on its surrounding provinces.

## 3. Materials and Methods

### 3.1. Variables

#### 3.1.1. Agricultural Green Development

Agricultural green development (*gad*) is the core variable in this paper. Based on a review of relevant literature in green agriculture, eco-agriculture, and organic agriculture [19,20,21], we know that agricultural green development focuses on the carrying capacity of resources and environment while also emphasizing the harmonization of economic, social, and ecological benefits. Therefore, we selected 14 indicators to measure the level of agricultural green development based on resource savings (*res*), environmental protection (*env*), ecological conservation (*eco*), and quality industrialization (*qua*), as shown in Table 1. Because of the overuse of pesticides, fertilizers, and plastic films at present, these three items are set as inverse indicators.

#### 3.1.2. Digital Inclusive Finance

The second core variable in this paper is digital inclusive finance (*dif*), which is specifically characterized by the total digital inclusive finance index at the provincial level in the Digital Inclusive Finance Index System of Peking University.

#### 3.1.3. Control Variables

Base on the main conclusion of the related literature [35,36], the following five indicators are selected as control variables in this paper: (1) financial agricultural input (*fai*) reflects the strength of state subsidies to agriculture and is measured by the share of expenditures on agriculture, forestry, and water affairs in local general public budget expenditures; (2) government investment in environmental management (*gep*) reflects the government’s investment in energy conservation and environmental protection and is expressed as the proportion of local general public budget expenditure on energy conservation and environmental protection; (3) the agricultural price index (*api*) reflects the price fluctuation of agricultural products and is expressed as the producer price index of agricultural products, of which the data for Tibet is missing and is replaced by its total regional production index [38]; (4) economic openness (*eor*) reflects the economic activity of the region and is defined as the proportion of total regional imports and exports to GDP; (5) the natural disaster incidence rate (*ndr*) reflects the natural production conditions of the region, and it is expressed as the proportion of the affected area to the total sown area of crops.

#### 3.1.4. Moderating Variables

Referring to Guo et al. [37], we select the level of education (*edu*), digital infrastructure (*inf*) and traditional finance (*rtf*) as the moderating variables. First, the level of education is expressed by the proportion of people with a college degree or above in the population aged 6 and above. Second, the digital infrastructure level is measured by the proportion of rural Internet access users. Third, the development level of rural traditions is expressed by the ratio of the year-end loan balance to the year-end deposit balance of rural credit cooperatives.

### 3.2. Data

Due to the incomplete data for Hong Kong, Macao, and Taiwan, the observed samples are limited to 31 provinces in China from 2011 to 2019. The sources of digital inclusive finance data from the Peking University Digital Inclusive Financial Index (2011–2020). The original data of agricultural green development can be seen in Appendix A. Except that the year-end loan balance and the year-end deposit balance of rural credit cooperatives are from the Wind Database, the original data of other control variables and moderating variables are from the China Statistical Yearbook.

### 3.3. Entropy-Weighted TOPSIS Method

The entropy-weight TOPSIS method is used to measure and evaluate agricultural green development in this paper. First, the data are dimensionless processed by the deviation method, then the index weight is determined by the entropy-weight method, and finally the relative level of agricultural green development in each province is calculated by the TOPSIS method. The steps of TOPSIS method are as follows:

Step 1: build a weighting matrix R for each evaluation index of agricultural green development according to the weights of each index calculated by the entropy weight method.

(1) $$R={\left(w{\;}_{j}\times Y_{ij}\right)}_{m\times n}$$

$w_{j}$ represents the weight calculated by entropy-weight method, $Y_{ij}$ represents the standardized value obtained by the dispersion method, $i\;\left(i=1,2,3,\ldots ,m\right)$ represents provinces, j(j=1, 2, 3,…,n) represents measurement indicators, m represents the number of provinces, n represents the number of measurement indicators.

Step 2: determine the positive ideal solutions $Q_{j}^{+}$ and negative ideal solutions $\;Q_{j}^{-}$.

(2) $$Q_{j}^{+}=\left(maxr_{i1},maxr_{i2},\cdots ,maxr_{im}\right)\;Q_{j}^{-}=\left(minr_{i1},minr_{i2},\cdots ,minr_{im}\right)$$

Step 3: calculate the distance measures for the positive ideal solutions $Q_{j}^{+}$ and negative ideal solutions $\;Q_{j}^{-}$.

(3) $$d_{i}^{+}=\sqrt{\overset{m}{\underset{j=1}{\sum }}{\left(Q_{j}^{+}-r_{ij}\right)}^{2}}\;d_{i}^{-}=\sqrt{\overset{m}{\underset{j=1}{\sum }}{\left(Q_{j}^{-}-r_{ij}\right)}^{2}}$$

Step 4: calculate the relative closeness to ideal solutions $C_{i}$.

(4) $$C_{i}=\frac{d_{i}^{-}}{d_{i}^{+}+d_{i}^{-}}$$

Here, $C_{i}\in \left[0,1\right]$. The higher $C_{i}$, the higher the level of agricultural green development in province i; and the smaller $C_{i}$, the lower the level of agricultural green development in province i.

### 3.4. Descriptive Statistics

The descriptive statistics of variables are shown in Table 2. The average value of agricultural green development is 0.428, and the standard deviation is 0.064. The standard deviation of digital inclusive finance is 0.679, which shows that digital inclusive finance varies greatly among provinces. The coefficient of variation of agricultural green development 0.150 is greater than that of digital inclusive finance 0.132, indicating that agricultural green development is more discrete. The mean values of the control variables, such as financial agricultural input, government investment in environmental management, agricultural price index, economic openness, and natural disasters incidence rate, are 0.151, 0.115, 0.030, 0.207, 0.234 and 5.143, respectively.

## 4. Econometric Model

### 4.1. Spatial Weight Matrix and Spatial Correlation

According to the first geographical law of Tobler [39], every place is connected. The reciprocal of the square of highway mileage $\;D_{ij}$ between cities is used to construct the geographical distance matrix, and the element $W_{ij}$ in row i and column j of the matrix is shown here:

(5) $$W_{ij}=\left\{\begin{array}{cc} 1/D_{ij}{}^{2} & i\neq j\\ 0 & i=j \end{array}\right.\;$$

Meanwhile, this paper uses the method of changing the spatial weight to test the robustness, and it uses the actual GDP of each province in the sample period to construct the economic distance matrix line  i number j column element $W_{ij}$. This expression is shown in Equation (6):

(6) $$W_{ij}=\left\{\begin{array}{cc} \frac{1}{D_{ij}{}^{2}}*diag\left(\frac{\bar{Y_{1}}}{\bar{Y}},\frac{\bar{Y_{2}}}{\bar{Y}},\frac{\bar{Y_{3}}}{\bar{Y}},\ldots ,\frac{\bar{Y_{i}}}{\bar{Y}}\right) & i\neq j\\ 0 & i=j \end{array}\right.$$

In Equation (6), $D_{ij}{}^{2}$ represents the square of the mileage of intercity roads, ${\bar{Y}}_{i}$ expresses the actual average GDP of the province i  in the sample period, and $\bar{Y}$ represents the average of the actual GDP of all provinces in the sample period.

This paper uses the Moran index to test the spatial correlation of variables, and the calculation formula is shown in Formula (7):

(7) $$Moran’s\;I=\frac{\sum_{i=1}^{n}\sum_{j=1}^{n}W_{ij}\left(Y_{i}-\bar{Y}\right)\left(Y_{j}-\bar{Y}\right)}{S^{2}\sum_{i=1}^{n}\sum_{j=1}^{n}W_{ij}}$$

$W_{ij}\;$ is the spatial weight matrix, $\bar{Y}\;$ is the agricultural green development.

### 4.2. Dynamic Spatial Durbin Model

In order to measure the spatial effect of digital inclusive finance on agricultural green development and take the continuity and spatial correlation of agricultural green development into account, the dynamic spatial Durbin model is chosen as the econometric model, and the expression is shown in Equation (8):

(8) $$\begin{array}{c} gad_{i,t}=c+\alpha_{1}gad_{i,t-1}+\beta_{1}\overset{n}{\underset{j=1}{\sum }}W_{ij}gad_{j,t}+\alpha_{2}dif_{i,t}+\beta_{2}\overset{n}{\underset{j=1}{\sum }}W_{ij}dif_{j,t}\\ \;\;\;\;\;\;\;+\alpha_{3}control_{i,t}+\beta_{3}W_{ij}control_{j,t}+\mu_{i}+\lambda_{t}+\varepsilon_{i,t} \end{array}$$

$gad_{j,t-1}$ represents the one-period lag of agricultural green development, $W_{ij}gad_{j,t}$ represents its spatial lag term, $W_{ij}dif_{j,t}$ indicates the effect of digital inclusive finance on agricultural green development in surrounding areas, and control represents the other control variables. $\alpha_{m}(m=1,\;2,\;3)$ and $\beta_{n}(n=1,\;2,\;3)$ are the regression coefficient, *c* is a constant term, *$\mu_{i}$*, $\lambda_{t}\;$ and $\varepsilon_{i,t}\;$ are individual effect, time effect, and random disturbance term, respectively. According to Lesage et al. [40], the spatial lag term makes the regression coefficient unable to accurately reflect the influence of dif  on gad, thus we use the effect decomposition to better describe the spatial interaction, in which the direct effect represents the influence of dif on this region, the indirect effect represents the influence of dif on other regions, and the total effect represents the average influence of dif on all regions.

From the setting process of spatial metrology model, we can see that dynamic spatial Durbin model can not only obtains short- and long-term spatial effects, but also investigates the lag term of variables. The spatial weight matrix $W_{ij}$ cannot be estimated, but it needs to be set in advance, which is a major disadvantage of spatial metrology.

Considering the regional heterogeneity, we further divide the sample into three subsamples, the eastern, middle, and western regions of China, to compare the heterogenous influence and spatial effect of digital inclusive finance on agricultural green development. According to the standards of the National Bureau of Statistics, the eastern region includes Beijing, Tianjin, Hebei, Liaoning, Shanghai, Jiangsu, Zhejiang, Fujian, Shandong, Guangdong, and Hainan; the middle region includes Shanxi, Jilin, Heilongjiang, Anhui, Jiangxi, Henan, Hubei, and Hunan; the western region includes the inner Mongolia Autonomous Region, the Guangxi Zhuang Autonomous Region, Chongqing, Sichuan, Guizhou, Yunnan, the Tibet Autonomous Region, Shaanxi, Gansu, Qinghai, the Ningxia Hui Autonomous Region, and the Xinjiang Uygur Autonomous Region.

### 4.3. Moderating Effect Model

Next, we build the moderating effect model to explore the heterogeneous effect of digital inclusive finance on regional agricultural green development in which the differences in the level of higher education, digital infrastructure, and rural traditional finance influence.

(9) $$\begin{array}{c} gad_{i,t}=c+\alpha_{1}gad_{i,t-1}+\beta_{1}\overset{n}{\underset{j=1}{\sum }}W_{ij}gad_{j,t}+\alpha_{2}dif_{i,t}+\beta_{2}\overset{n}{\underset{j=1}{\sum }}W_{ij}M\times dif_{j,t}\\ \;\;\;\;\;\;\;+\beta_{3}control_{i,t}+\alpha_{3}W_{ij}control_{j,t}+\mu_{i}+\lambda_{t}+\varepsilon_{i,t} \end{array}$$

M  represents the dummy variables after moderating variables (*edu*, *inf* or *rtf*) are assigned, when $\;M=\left\{\begin{array}{c} 1\;M_{i}>means\left(M_{1},M_{2},\cdots ,M_{i-1}\right)\\ \;0\;\;M_{i}\leq means\left(M_{1},M_{2},\cdots ,M_{i-1}\right)\; \end{array}\right.$. Other variables are used similarly to Equation (8).

## 5. Empirical Results

### 5.1. Stationary Test and Spatial Correlation Test

Before conducting the regression analysis in this paper, the Levin, Lin and Chu (2002) Panel Unit Root Test (LLC) and Im, Pesaran and Shin (2002) Panel Unit Root Test (IPS) tests are used to determine the stationarity of variables. The results show that all variables pass the LLC tests at the significance level of 1%, while the variables pass the IPS tests at least at the 5% significance level. All the variables are stable, as shown in Table 3.

The global Moran’s I, an important indicator to judge the spatial correlation of variables [41], is performed to test agricultural green development and digital inclusive finance of 31 provinces in China from 2011 to 2019. As can be seen in Table 4, the results for agricultural green development and digital inclusive finance are positive at the significance level of 1%, indicating that both have significant positive spatial autocorrelation.

**Figure 1:**
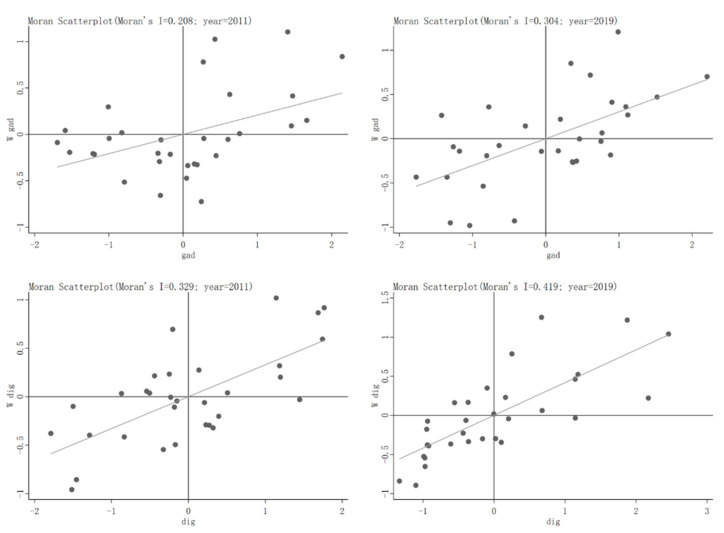
Local Moran’s I index of digital inclusive finance and agricultural green development.

To further investigate the spatial dependence of specific regions, we use the local Moran’s I to analyze the spatial relationship between each region and adjacent areas. The results are shown in Figure 1. From the local Moran’s I scatter diagram distribution, it can be seen that the agricultural green development and digital inclusive finance are basically concentrated in the first and third quadrants, showing the characteristics of HH and LL agglomeration. The result of the local Moran’s I index is the same as the test result of global Moran’s I index, indicating that both have obvious positive spatial correlation.

### 5.2. Analysis of the Baseline Empirical Results

Table 5 shows the results of the effect of digital inclusive finance on agricultural green development; Columns 2 to 4 represent the estimation results of the samples in the east, middle, and west, respectively.

As shown in Column 1, digital inclusive finance effectively promotes agricultural green development, with the promotion effect being a temporary and spatial spillover. In terms of the direct effect of digital inclusive finance on agricultural green development, the estimated coefficient in the short term is 0.189 while its long-term direct effect is positive but insignificant, suggesting that digital inclusive finance can promote agricultural green development; however, its positive effect is only temporary. One possible reason for this is that a large injection of digital inclusive finance will leverage rapid agricultural green development in the short term. However, as the conflict between the government’s goal of supporting agriculture and market institutions’ profit intensifies, the goal of digital inclusive finance in rural areas may not be achieved [42]. Thus, this effect is weakened in the long term.

In terms of indirect effects, the coefficients in the short-term and long-term effects are positive and pass the 1% significance test in the short term, but not in the long term. This suggests that there is a positive spatial spillover of digital inclusive finance on agricultural green development in the short term, effectively demonstrating the green behavior of agricultural production in neighboring provinces. However, the long-term indirect effect is not significant. One possible reason for this that the spatial spillover effect of digital inclusive finance has to be realized through technological innovations such as the Internet [43]. The spillover radiation of technological innovations gradually shrinks with time, weakening the spillover effect of digital inclusive finance [44].

In addition, both the time-lagged term and the space-lagged term of agricultural green development are positively significant at the 1% level, indicating superimposed and spatial spillover effects.

Second, the short-term positive effect of digital inclusive finance on agricultural green development existing in the eastern, middle, and western regions of China also experiences spatial spillover. Specifically, the short-term positive effect is most obvious in the middle, where spatial spillover also performs better. The reasons are as follows. The development level of digital inclusive finance in the middle region is on a higher level than the other regions. As the main agricultural producing areas, the middle faces the greatest pressure of resource conservation and environmental constraints [45]. Thus, digital inclusive finance for agricultural green development can be more easily adopted, and it has high potential for agricultural green development.

Third, the role of digital inclusive finance in supporting agricultural green development in the western region is limited in the long run. Due to the level of digital inclusive finance development as well as technological development and innovation, digital inclusive finance cannot play a larger leveraging role in promoting agricultural green development in the western region [37]. However, spatial spillover is not significant in all three regions, possibly for the following reasons. The current development of digital inclusive finance in rural areas is still in its infancy [6] and has not yet formed a stable and sustained driving effect on the green development of agriculture in neighboring provinces.

### 5.3. Analysis of Moderating Results

Columns 1 to 3 of Table 6 show the moderating results of the effect of digital inclusive finance on agricultural green development after adding the dummy variables’ interaction with digital inclusive finance and education, digital infrastructure, and traditional finance.

First, digital inclusive finance can provide basic financial services for groups with low education levels and enhance its role in promoting agricultural green development, but its spatial spillover effect on agricultural green development is not affected by education. Specifically, the short- and long-term direct effects are significantly positive, and the long-term direct effect is greater than the short-term direct effect. This shows that the positive effect of digital inclusive finance on agricultural green development is greater in provinces with lower education levels, and this phenomenon grows more obvious with time. Meanwhile, the short- and long-term indirect effects are not significant, indicating that education does not affect the spatial spillover effect.

Second, digital inclusive finance has greater support and spatial spillover effect on agricultural green development in provinces with low levels of digital infrastructure. The estimation coefficients of short- and long-term direct effects are 0.006 and 0.020, respectively, both at the 5% significant level. The long-term direct effect is greater than the short-term direct effect, which indicates that the provinces with lower digital infrastructure levels have stronger support for agricultural green development. This phenomenon is more obvious with the passage of time. The reasons for this are as follows. At the initial stage, the digital inclusive finance mostly depended on offline promotion, and its supporting role was not greatly limited by the level of digital infrastructure [13]. With the gradual improvement of digital infrastructure, the development of digital inclusive finance in provinces with backward digital infrastructure has become more rapid. Thus, its long-term role is more significant. Meanwhile, the short- and long-term indirect effects are significantly positive, indicating that digital infrastructure affects the spatial spillover effect: the lower the digital infrastructure, the greater the spatial spillover effect.

Third, the low level in traditional finance can restrain the short-term effect of digital inclusive finance on agricultural green development. In the long term, the emergence of late-developing advantages will enhance the effect. The short-term direct effect is significantly negative, while the long-term direct effect is positive but insignificant. This phenomenon indicates that the short-term positive effect of digital inclusive finance on agricultural green development is more obvious in provinces with higher levels of traditional finance; however, this advantage of such provinces will be gradually lost in the long term, for the following potential reasons. The role of digital inclusive finance relies on traditional finance in the early stages of development [37]. Meanwhile, the short-term indirect effect is significantly negative, and the long-term indirect effect is not significant, suggesting that the spatial spillover effect also depends on traditional finance in the short term.

### 5.4. Robustness Test

The robustness test results are shown in Columns 1 to 4 in Table 7. As can be seen, the regression results based on the economic distance weight matrix do not differ from those based on the time distance weight matrix above. The significant levels of the direct, indirect, and total effects of digital inclusive finance on agricultural green development are consistent, indicating that the estimation results are robust to a certain extent.

Table 8 shows the robustness test results of the moderating effect. Columns 1 to 3 show the effect of digital finance on agricultural green development in provinces with different moderating variables after changing the spatial weight. The results show that the significant level of interaction items is consistent with Table 8, indicating its robustness.

## 6. Conclusions and Policy Implications

### 6.1. Conclusions

This paper selects 14 indicators to construct agricultural green development from four dimensions of resource savings, environmental protection, ecological conservation, and quality industrialization using provincial panel data from 2011 to 2019. Then, agricultural green development is estimated using the entropy-weighted TOPSIS method. By using the dynamic spatial Durbin model, we empirically analyze the effect of digital inclusive finance on agricultural green development. Finally, we choose education, digital infrastructure, and traditional finance as moderating variables to explore this effect.

The dynamic SDM results show that digital inclusive finance has a positive effect on agricultural green development, and the short-term effect is more significant than the long-term effect, suggesting that the promotion of digital inclusive finance could be an effective measure for ensuring agricultural modernization. Furthermore, digital inclusive finance has regional heterogeneous effects on agricultural green development. Specifically, in the short term, the direct and indirect effects of digital inclusive finance on agricultural green development are more pronounced in the middle region, while in the long term, both effects on agricultural green development are only effective in the eastern region.

We find that digital inclusive finance also exhibits heterogeneity in agricultural green development in provinces with different socio-economic characteristics, such as education, digital infrastructure, and traditional finance. Higher levels of the above three indicators tend to result in greater and more significant effects on the level of digital inclusive finance.

Due to the impact of COVID-19, the research data collection period is limited. At the same time, this research is based on China’s national conditions, and other developing countries or developed countries can be selected for future research.

### 6.2. Policy Implications

According to the conclusions, the following policy implications should be put forward:

(1) Promoting the overall development of digital inclusive finance. Digital inclusive finance can effectively promote agricultural green development and has regional heterogeneity. Therefore, the government should provide legal and financial support to financial institutions, ensuring the quantity and level of financial supply in rural areas, and pay attention to digital technology innovation.

(2) Focusing on the sustainability of the development of digital inclusive finance. The east should give better play to the spillover effect and support the development of the middle and western regions. The middle should accelerate the transformation of agricultural industrialization and carbon reduction. Due to the restriction of natural resources and different industrial forms, the west should actively use digital technology to guide financial resources to flow to agricultural technology innovation activities.

(3) Improving the financial literacy and skills of rural people. Digital finance education and training is an important way to improve the financial literacy and skills of rural people. The improvement of financial literacy is conducive to farmers’ mastering financial knowledge. It is suggested to provide diversified and multi-channel financial education and training for rural areas.

(4) Strengthening rural digital financial infrastructure. Digital inclusive finance is limited by digital financial infrastructure. Therefore, the government should vigorously promote the construction of rural digital financial infrastructure, formulate corresponding preferential policies in combination with the development of regional characteristics, encourage financial institutions to take root in rural development, and further release the positive role of digital inclusive finance.

(5) Promoting the innovative combination of traditional finance and digital technology to develop digital inclusive finance. Such measures can build financial and agricultural service platforms through innovating financial instruments, and provide financial support for farmers’ activities, such as agricultural carbon reduction production, living and production pollution prevention, and ecological protection.

## Figures and Tables

**Table 1:** The system of the agricultural green development index.

agricultural green development	**Dimensions**	**Indicators**	**Unit**	**Index Type**	**Variable Definition**	**Means**	**S.D.**
	resource savings	multiple cropping index of cropland	%	+	improve the intensity of land resource utilization	1.253	0.376
		water saving irrigation cropland ratio	%	+	improve the utilization intensity of water resources	0.291	0.231
		land labor ratio	ha/num	+	increase labor input intensity	5.833	3.278
		use intensity of agricultural machinery	kw/ha	+	improve resource utilization intensity	8.154	3.566
	environmental protection	use intensity of pesticide	kg/ha	−	reduce pesticide use intensity	15.316	12.540
		use intensity of chemical fertilizer	kg/ha	−	reduce the use intensity of chemical fertilizer	462.284	212.737
		use intensity of diesel	kg/ha	−	reduce agricultural waste discharge	210.862	208.644
		use intensity of plastic film	kg/ha	−	reduce agricultural waste discharge	23.247	16.974
	ecological conservation	area ratio of nature reserves	%	+	strengthen the protection of natural ecological environment	0.117	0.122
		area ratio of wetland	%	+	strengthen wetland environmental protection	0.086	0.124
		forest coverage	%	+	improve ecological conservation	0.328	0.180
	quality industrialization	amount of green food labeling enterprises per unit cropland area	num/10,000 ha	+	improve the industrialization of green agriculture	0.576	1.588
		amount of green food labeling products per unit cropland area	num/10,000 ha	+	improve the brand quality of green agricultural products	1.194	2.648
		agricultural income per unit cropland area	10,000 yuan/ha	+	increase farmers’ agricultural income	4.837	2.589

**Table 2:** Descriptive statistics of variables.

Variable	Means	S.D.	C·V	Minimum	Maximum
*g* *ad*	0.428	0.064	0.150	0.318	0.751
*dif*	5.143	0.679	0.132	2.786	6.017
*fai*	0.115	0.033	0.287	0.041	0.203
*gep*	0.030	0.010	0.333	0.012	0.068
*api*	0.207	0.113	0.546	0.016	0.900
*eor*	0.234	0.280	1.197	0.005	1.441
*ndr*	0.151	0.117	0.775	0.000	0.618
*edu*	0.135	0.073	0.541	0.024	0.505
*inf*	0.222	0.112	0.505	0.000	0.596
*rtf*	0.472	0.127	0.269	0.188	0.814

**Table 3:** Variable stationarity test.

Variable	LLC	IPS
*gad*	−35.464 ***	−10.001 ***
*dif*	−20.414 ***	−14.657 ***
*fai*	−18.212 ***	−4.381 ***
*gep*	−12.799 ***	−1.558 ***
*api*	−24.460 ***	−5.678 ***
*eor*	−27.708 ***	−9.875 ***
*ndr*	−16.073 ***	−3.770 ***

Notes: *** denote statistical significance levels at 1%.

**Table 4:** Global Moran’s I index of digital inclusive finance and agricultural green development.

Year	*gad*	*dif*
2011	0.220 ***	0.217 ***
2012	0.290 ***	0.267 ***
2013	0.239 ***	0.261 ***
2014	0.275 ***	0.272 ***
2015	0.267 ***	0.251 ***
2016	0.285 ***	0.262 ***
2017	0.328 ***	0.288 ***
2018	0.273 ***	0.315 ***
2019	0.256 ***	0.320 ***

Notes: *** denote statistical significance levels at 1%.

**Table 5:** The results of the dynamic spatial Durbin model.

		Pool	Subsample		
		China	East	Middle	West
		(1)	(2)	(3)	(4)
$L.gad_{t-1}$		1.157 ***	0.904 ***	0.957 ***	1.352 ***
		(0.015)	(0.038)	(0.022)	(0.032)
short-term	Direct effect	0.189 ***	0.556 ***	0.635 ***	0.284 ***
		(0.036)	(0.140)	(0.092)	(0.069)
	Indirect effect	1.040 ***	1.527 ***	1.587 *	0.886 *
		(0.124)	(0.268)	(0.207)	(0.403)
	Total effect	1.229 ***	2.083 ***	2.222 ***	1.070 ***
		(0.125)	(0.328)	(0.235)	(0.457)
long-term	Direct effect	0.117	0.067 ***	0.014 **	0.051
		(0.641)	(0.067)	(0.017)	(0.057)
	Indirect effect	0.783	0.312	0.048	0.062
		(0.083)	(0.068)	(0.048)	(0.062)
	Total effect	0.056	0.146 **	0.249	1.079
		(0.031)	(0.063)	(0.027)	(0.196)
Control variables		Yes			
ρ		0.277 ***	0.192 ***	0.058 ***	0.543 ***
$R^{2}$		0.987	0.995	0.998	0.932
$\sigma^{2}$		0.001 ***	0.001 ***	0.001 ***	0.001 ***
L-L		249.700	233.500	378.200	272.400

Notes: $gad_{j,t-1}$ represents the one-period lag of agricultural green development. *t* statistics in parentheses. ρ represents the spatial autoregressive coefficient. L-L represents the log likelihood estimator. *, ** and *** denote statistical significance levels at 10%, 5%, and 1%, respectively.

**Table 6:** The results of the moderating effect model.

		Education	Digital Infrastructure	Traditional Finance
		(1)	(2)	(3)
$L.gad_{t-1}$		1.144 ***	1.177 ***	1.151 ***
		(0.015)	(0.015)	(0.015)
short-term	Direct effect	0.008 **	0.006 **	−0.002 **
		(0.003)	(0.003)	(0.003)
	Indirect effect	0.014	0.007 *	−0.005 *
		(0.001)	(0.004)	(0.003)
	Total effect	0.007 **	0.007 *	−0.006 **
		(0.004)	(0.004)	(0.003)
long-term	Direct effect	0.029 **	0.020 **	0.004 *
		(0.006)	(0.034)	(0.184)
	Indirect effect	0.016	0.008 *	0.018
		(0.003)	(0.035)	(0.185)
	Total effect	0.015 **	0.013 *	0.014 *
		(0.007)	(0.006)	(0.007)
Control variables		Yes		
ρ		0.231 ***	0.223 ***	0.203 ***
$R^{2}$		0.937	0.933	0.940
$\sigma^{2}$		0.001 ***	0.001 ***	0.001 ***
L-L		261.040	226.274	260.835

Notes: $gad_{j,t-1}$ represents the one-period lag of agricultural green development. *t* statistics in parentheses. ρ represents the spatial autoregressive coefficient. L-L represents the log likelihood estimator. *, ** and *** denote statistical significance levels at 10%, 5%, and 1%, respectively.

**Table 7:** Robustness test results for the dynamic spatial Durbin model.

		Pool	Subsample		
		China	East	Middle	West
		(1)	(2)	(3)	(4)
$L.gad_{t-1}$		1.113 ***	1.055 ***	0.994 ***	1.582 ***
		(0.015)	(0.036)	(0.022)	(0.039)
short-term	Direct effect	0.154 ***	0.266 ***	0.432 ***	0.268 ***
		(0.034)	(0.152)	(0.168)	(0.207)
	Indirect effect	0.923 ***	1.112 ***	1.688 *	0.187 *
		(0.163)	(0.386)	(0.446)	(0.060)
	Total effect	1.077 ***	1.544 ***	1.954 ***	1.404 ***
		(0.159)	(0.458)	(0.588)	(0.587)
long-term	Direct effect	0.126	0.451 ***	0.329 **	0.151
		(0.007)	(0.161)	(0.451)	(0.413)
	Indirect effect	0.045	0.121	0.047	0.019
		(0.004)	(0.121)	(1.120)	(0.019)
	Total effect	0.103	0.246 **	1.679	0.133
		(0.339)	(0.093)	(1.234)	(0.204)
Control variables		Yes			
ρ		0.297 ***	0.358 ***	0.513 ***	0.478 ***
$R^{2}$		0.989	0.994	0.895	0.989
$\sigma^{2}$		0.001 ***	0.001 ***	0.001 ***	0.001 ***
L-L		280.400	242.612	292.641	218.600

Notes: $gad_{j,t-1}$ represents the one-period lag of agricultural green development. *t* statistics in parentheses. ρ represents the spatial autoregressive coefficient. L-L represents the log likelihood estimator. *, ** and *** denote statistical significance levels at 10%, 5%, and 1%, respectively.

**Table 8:** Robustness test results of the moderating effect model.

		Education	Digital Infrastructure	Traditional Finance
		(1)	(2)	(3)
$L.gad_{t-1}$		1.050 ***	1.130 ***	1.153 ***
		(0.015)	(0.015)	(0.015)
short-term	Direct effect	0.006 **	0.004 **	−0.002 **
		(0.001)	(0.001)	(0.001)
	Indirect effect	0.011	0.002 *	−0.005 *
		(0.004)	(0.003)	(0.003)
	Total effect	0.005 **	0.002 *	−0.006 **
		(0.004)	(0.004)	(0.003)
long-term	Direct effect	0.023 **	0.012 **	0.011 *
		(0.711)	(0.004)	(0.004)
	Indirect effect	0.030	0.007 *	0.027
		(0.710)	(0.006)	(0.002)
	Total effect	0.013 **	0.005 *	0.013 *
		(0.011)	(0.009)	(0.007)
Control variables		Yes		
ρ		0.249 ***	0.191 ***	0.213 ***
$R^{2}$		0.955	0.953	0.943
$\sigma^{2}$		0.001 ***	0.001 ***	0.001 ***
L-L		368.823	326.840	279.574

Notes: $gad_{j,t-1}$ represents the one-period lag of agricultural green development. *t* statistics in parentheses. ρ represents the spatial autoregressive coefficient. L-L represents the log likelihood estimator. *, ** and *** denote statistical significance levels at 10%, 5% and 1%, respectively.

## Data Availability

Data supporting the conclusions of this article are included within the article. The dataset presented in this study are available on request from the corresponding author.

## Appendix A. Index Measurement and Original Data Source of Agricultural Green Development

agricultural green development	**Indicators**	**Measurements**	**Data Sources**
	multiple cropping index of cropland	$\frac{sown\;area\;of\;crops}{cropland\;area}$	sown area of crops from the China Statistical Yearbook; cropland area from the China Statistical Yearbook, and this data below in the same column of the table is the same
	water saving irrigation cropland ratio	$\frac{water\;saving\;irrigation\;cropland}{cropland\;area}$	water saving irrigation cropland from the China Environmental Yearbook
	land labor ratio	$\frac{cropland\;area}{rural\;population}$	rural population from the China Statistical Yearbook
	use intensity of agricultural machinery	$\frac{usage\;of\;agricultural\;machinery}{cropland\;area}$	usage of agricultural machinery from the China Rural Statistical Yearbook
	use intensity of pesticide	$\frac{usage\;of\;pesticide}{cropland\;area}$	usage of pesticide from the China Rural Statistical Yearbook
	use intensity of chemical fertilizer	$\frac{usage\;of\;chemical\;fertilizer}{cropland\;area}$	usage of chemical fertilizer from the China Rural Statistical Yearbook
	use intensity of diesel	$\frac{usage\;of\;diesel}{cropland\;area}$	usage of diesel from the China Rural Statistical Yearbook
	use intensity of plastic film	$\frac{usage\;of\;plastic\;film}{cropland\;area}$	usage of plastic film from the China Rural Statistical Yearbook
	area ratio of nature reserves	-	the China Statistical Yearbook
	area ratio of wetland	-	the China Statistical Yearbook
	forest coverage	-	the China Statistical Yearbook
	amount of green food labeling enterprises per unit cropland area	$\frac{amount\;of\;green\;food\;labeling\;enterprises}{cropland\;area}$	amount of green food labeling enterprises from the China Green Food Network
	amount of green food labeling products per unit cropland area	$\frac{amount\;of\;green\;food\;labeling\;products}{cropland\;area}$	amount of green food labeling products from the China Green Food Network
	agricultural income per unit cropland area	$\frac{agricultural\;income}{cropland\;area}$	agricultural income from the China Statistical Yearbook
